# Regulation of DNA phosphorothioate modification in *Salmonella enterica* by DndB

**DOI:** 10.1038/srep12368

**Published:** 2015-07-20

**Authors:** Wei He, Teng Huang, You Tang, Yanhua Liu, Xiaolin Wu, Si Chen, Wan Chan, Yajie Wang, Xiaoyun Liu, Shi Chen, Lianrong Wang

**Affiliations:** 1 Key Laboratory of Combinatorial Biosynthesis and Drug Discovery, Ministry of Education, and School of Pharmaceutical Sciences, Wuhan University, Wuhan 430071, China; 2 Taihe Hospital, Hubei University of Medicine, Shiyan, Hubei, China; 3 Institute of Analytical Chemistry and Synthetic and Functional Biomolecules Center, College of Chemistry and Molecular Engineering, Peking University, Beijing 100871, China; 4 Department of Chemistry, The Hong Kong University of Science and Technology, Clear Water Bay, Kowloon, Hong Kong, China

## Abstract

DNA phosphorothioate (PT) modification, in which the non-bridging oxygen of the sugar-phosphate backbone is substituted by sulfur, occurs naturally in diverse bacteria and archaea and is regulated by the DndABCDE proteins. DndABCDE and the restriction cognate DndFGHI constitute a new type of defense system that prevents the invasion of foreign DNA in *Salmonella enterica* serovar Cerro 87. GAAC/GTTC consensus contexts across genomes were found to possess partial PT modifications even in the presence of restriction activity, indicating the regulation of PT. The abundance of PT in cells must be controlled to suit cellular activities. However, the regulatory mechanism of PT modification has not been characterized. The result here indicated that genomic PT modification in *S. enterica* is controlled by the transcriptional regulator DndB, which binds to two regions in the promoter, each possessing a 5′-TACGN^10^CGTA-3′ palindromic motif, to regulate the transcription of *dndCDE* and its own gene. Site-directed mutagenesis showed that the Cys29 residue of DndB plays a key role in its DNA-binding activity or conformation. Proteomic analysis identified changes to a number of cellular proteins upon up-regulation and loss of PT. Considering the genetic conservation of *dnd* operons, regulation of PT by DndB might be widespread in diverse organisms.

Phosphorothioate (PT) modification is a unique physiological modification in the sugar-phosphate backbone of DNA, in which the non-bridging oxygen is replaced by sulfur in a sequence-selective and *R*_P_ stereo-specific manner by Dnd proteins^1^. The five-gene *dnd* cluster was originally identified by electrophoresis to cause a DNA degradation phenotype in *Streptomyces lividans.* The cluster has since been detected in more than 200 bacterial and archaeal strains and shows a highly conserved genetic organization^2^,^3^. Some *dnd* clusters consists of only *dndBCDE* without a linked *dndA.* DndA is a NifS-like cysteine desulfurase that can be functionally replaced by an IscS homolog located elsewhere in the genome^4^,^5^. *dndFGHI* is a divergently transcribed cluster that was later identified in the vicinity of the *dndBCDE* operon. The DNA PT system shows similarity to the methylation-based restriction-modification (RM) system; DndABCDE enables the PT modification of ‘self’ DNA, and the restriction cognate DndFGHI discriminates and destroys non-labeled foreign DNA via double-stranded DNA breakage in *S. enterica* serovar Cerro 87^6^,^7^. A subset of *dndBCDE* clusters is not accompanied by *dndFGHI* in diverse strains. This is reminiscent of an orphan DNA methyltransferase, implying that the DNA PT system plays other physiological roles in addition to host protection^8^,^9^.

The DNA PT system is more intricate than typical RM systems and involves five Dnd proteins that sequence-selectively and *R*_P_ stereo-specifically incorporate sulfur. The DndA protein is capable of assembling DndC as a 4Fe-4S cluster protein, and DndC shows significant homology to phosphoadenosine phosphosulfate (PAPS) reductase^10^. DndB is a functionally unknown protein with a conserved DGQHR sequence motif, and DndD exhibits ATPase activity and has been proposed to introduce nicks in DNA during sulfur incorporation^11^. The DndE protein adopts a tetramer conformation and has binding affinity for nicked double-stranded DNA *in vitro* via positively charged lysine residues on its surface^12^. However, lysine residue mutations do not have a significant effect on the total PT modification frequency under physiological conditions, implying that the dsDNA binding capacity of DndE may not be crucial for PT modification and/or that DndE may have other biological functions^13^. As a newly identified component of the PT system, DndFGHI in *S. enterica* serovar Cerro 87 has been found to induce double-stranded DNA breaks in non-PT-modified DNA^6^,^14^.

The replacement of an oxygen atom with sulfur confers the PT-modified DNA backbone with resistance to nucleases. Upon DNA hydrolysis, PT-linked dinucleotides are generated in addition to canonical mononucleotides^1^. This phenomenon can be detected by a sensitive liquid chromatography-coupled tandem quadrupole mass spectrometry (LC-MS/MS) method that can be used to identify PT sequence contexts and quantify PT frequencies^15^. PT-modified d(G_PS_A) and d(G_PS_T) have been identified in *Escherichia coli* B7A, which has Dnd proteins highly homologous to those in *S. enterica* serovar Cerro 87, at frequencies of 370 ± 11 and 398 ± 17 per 10^6^ nt, whereas a frequency of 2,624 ± 22 of d(C_PS_C) per 10^6^ nt has been detected in *Vibrio cyclitrophicus* FF75^15^. Our recent application of single-molecule, real-time (SMRT) sequencing to characterize genomic PT sites has revealed unique features of DNA PT systems^16^. The d(G_PS_A) and d(G_PS_T) sites in *E. coli* B7A are located within stringently conserved 4-bp G_PS_AAC/G_PS_TTC sequences. However, only 12% (4855) of 40,701 GAAC/GTTC sites on the *E. coli* B7A chromosome demonstrate PT modifications, even in the presence of active DndFGHI. In contrast, PT mapping in *V. cyclitrophicus* FF75 revealed single-stranded PT modifications in C_PS_CA motifs. The same finding of incomplete modification has been observed for *V. cyclitrophicus* FF75, in which only 14% of genomic CCA sites have undergone PT modifications^16^. PT modifications have been found to be distributed across the *E. coli* B7A and *V. cyclitrophicus* FF75 chromosomes without an apparent preference for open reading frames, tRNA, rRNA or noncoding regions^16^. Unexpectedly, partial PT modifications have been observed at given sites in the population of DNA molecules in both *E. coli* B7A and *V. cyclitrophicus* FF75^16^. These highly unusual features differentiate DNA PT systems from typical RM systems and known DNA epigenetic mechanisms.

The PT linkage in the DNA backbone is susceptible to cleavage by an oxidative Tris derivative generated in the electrophoretic buffer adjacent to the anode, resulting in a DNA degradation (Dnd) phenotype^17^. The disruption of *dndA*, *dndC*, *dndD* or *dndE* abolishes the degradation phenotype, whereas the mutation of *dndB* preserves the Dnd phenomenon in both *S. enterica* serovar Cerro 87 and *Streptomyces lividans* 1326^5^. The levels of genomic PT modifications vary from 3–8 per 10^6^ nt to 2-3 per 10^3^ nt in diverse bacterial strains^15^. The low abundance of PT suggests that the modification must be controlled to suit cellular activities. The regulation of PT modification is important for understanding its cellular importance. However, the molecular mechanism of PT regulation has not been characterized.

Given the ubiquity of DNA PT systems, we decided to perform a detailed investigation of how genomic PT modifications are regulated. In this study, we first observed a 2-fold increased PT frequency in response to *dndB* deletion in *S. enterica* serovar Cerro 87. Subsequent proteomic analysis revealed that DndCDE was the most up-regulated protein in response to the deletion of *dndB*. DndB, a transcriptional regulator, binds to its promoter region to influence the transcription of *dndCDE* and its own gene to regulate the frequency of genomic PT modification. Considering the genetic conservation of *dndBCDE* operons, regulation of PT frequency by DndB might be widely adopted in diverse organisms.

## Results

### PT modifications in *dnd* mutants

The disruption of individual *dndCDE* genes completely abolished the DNA degradation phenotype, whereas disruption of *dndB* in *S. lividans* and *S. enterica* serovar Cerro 87 led to smeared DNA^9^,^18^. These results prompted us to first compare the PT modifications in wild-type *S. enterica* serovar Cerro 87 and individual *dnd* mutants using sensitive LC-MS/MS. The results of these quantifications were well-correlated with the electrophoretic phenotype, with no DNA PT modifications detected in the *dndCDE* mutants. In contrast, *dptB*^***−***^, the *dndB* in-frame deletion mutant, still carried PT modifications. Moreover, the total PT modifications in *dptB*^***−***^ occurred at a frequency of 1158 ± 192 per 10^6^ nt, which is twice the rate measured in wild-type *S. enterica* serovar Cerro 87 (594 ± 11 per 10^6^ nt) (Table 1). However, the PT modifications in *dptB*^***−***^ still strictly occurred at d(G_PS_A) and d(G_PS_T) sites at a 1:1 ratio, suggesting that the preference for the G_PS_AAC/G_PS_TTC consensus sequence was unaltered.

### Global proteome analysis of *dnd* mutants

**Figure 1:**
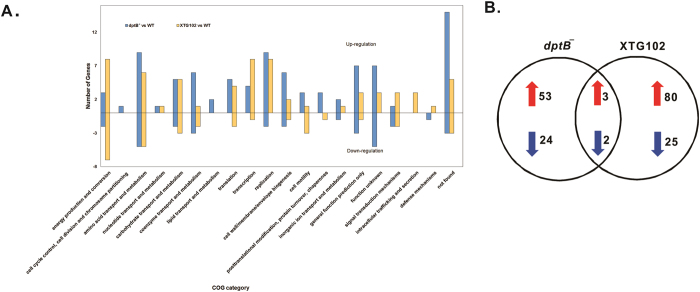
Comparison of gene expression data in *dptBˉ* and XTG102. (**A**) COG distribution of differentially expressed proteins in *dptBˉ* and XTG102. Each bar represents the actual number of proteins differentially expressed with a fold change of higher than 1.5 or lower than 0.66 (*p*-value < 0.05). Because the COG annotation groups overlap, the sum of COG annotated genes is larger than the number of total up- and down-regulated genes analyzed. (**B**) Venn diagram of common and differentially expressed genes in *dptBˉ* and XTG102. Red and blue arrows indicate up- or down-regulated genes.

To understand the molecular basis of altered PT abundance and the global impact of excessive PT on cellular physiology, proteomic analysis was carried out to identify proteins that were differentially expressed in *dptB*^***−***^ and XTG102, a *dndBCDE* deletion mutant without PT. In total, 82 and 110 proteins were differentially expressed with a fold change higher than 1.5 or lower than 0.66 (*p*-value < 0.05) in *dptB*^***−***^ and XTG102 relative to wild-type *S. enterica* serovar Cerro 87 (GenBank no. CP008925) (Supplementary Table S2). The 82 and 110 proteins were classified into 19 and 18 clusters of orthologous groups (COGs), respectively, based on their predicted function (Fig. 1).

In *dptB*^***−***^ cells, DndC (18.8-fold), DndD (9.5-fold) and DndE (15.4-fold) were induced to the greatest extents (Supplementary Table S2). In contrast, no significant changes in the restriction cognates DndFGHI were observed in *dptB*^***−***^ following the increase in total PT. However, five genes in the replication group—the DNA gyrase GyrA, the DNA mismatch repair (MMR) protein MutS, the nucleotide excision repair (NER) protein UvrA, topoisomerase IV subunit B and DNA polymerase I—were activated in *dptB*^***−***^ but not in XTG102 (Supplementary Table S2, Fig. 1). The transcription category represented another significant fraction of the differentially expressed members of the proteome. Four transcriptional regulators—MalT (GW13_PRO2528), NanR (GW13_PRO2351), TorR (GW13_PRO2606), and a GntR family regulator (GW13_PRO2829) —as well as a transcription associated protein (GW13_PRO2516) and an RNA polymerase associated protein (RapA; GW13_PRO3646), were significantly up-regulated. More than half of the 26 down-regulated proteins were categorized into the energy production and conversion group or the amino acid transport and metabolism groups.

In response to PT loss in XTG102, the most noticeable change was the activation of a number of SOS genes (e.g., *recA*, *dinI*, *radA*, *ruvA*, *ruvB*, *rmuC*, *yebG*, *gyrI*), as well as eight prophage genes (i.e., GW13_PRO0152, GW13_PRO0162, GW13_PRO0165, GW13_PRO0175, GW13_PRO0176, GW13_PRO0183, GW13_PRO0191, and GW13_PRO0194) (Supplementary Table S2). In addition, the expression levels of nine genes involved in replication (e.g., *recN*, *ruvA*, *ruvB*, *recA*) were elevated, many of which overlapped with the SOS genes. These features were similar to those observed in the transcriptomic profile of DNA without PT protection that suffered double-stranded cleavage and then triggered the SOS response and prophage induction^6^. The comparison of gene expression data in *dptB*^***−***^ and XTG102 showed distinctive proteomic profiles. As shown in Fig. 1B, only five proteins were commonly up-regulated: GW13_PRO2474, GW13_PRO2275, and GW13_PRO0191, a phosphoribulokinase homolog, a phosphoheptose isomerase and a hypothetical protein. The commonly down-regulated proteins were DndB and GW13_PRO3998, a putative oxidoreductase.

### DndB binds to the promoter region of the *dnd* operon

**Figure 2:**
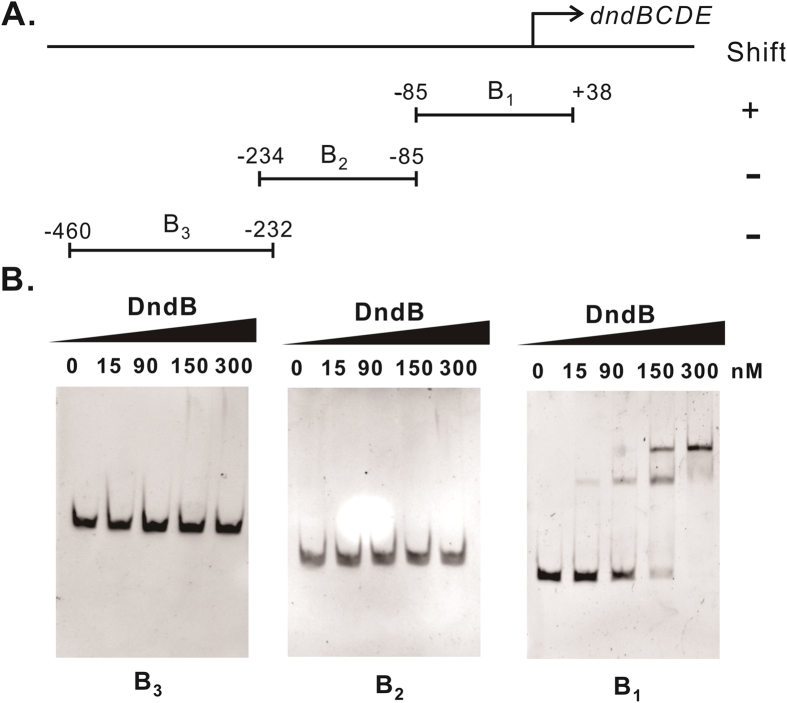
DndB binding sites in the *dndBCDE* promoter region. (**A**) Localization of the DNA fragments used in the EMSAs. The numbers represent the positions of the fragments with respect to the transcription start site. (**B**) EMSAs were performed using serial concentrations of DndB ranging from 0 to 300 nM with the DNA fragments B_3_, B_2_ and B_1_.

Up-regulation of the DndCDE proteins and increased PT frequencies in *dptB*^***−***^ implied that DndB might regulate genomic PT by influencing the transcription of the *dnd* genes. We first compared the transcript levels of *dndCD* by quantitative RT-PCR and observed a dramatic increase of approximately 15-fold after *dndB* deletion. Complementing the *dptB*^***−***^ mutation with a plasmid (pWHU746) containing *dndB* lowered the transcription of *dndCD* and decreased the total PT to 632 ± 12 per 10^6^ nt (Table 1). EMSAs were performed to assess the direct interaction of the DndB protein with its promoter DNA. The native mass of purified DndB was determined to be 79 kDa by size exclusion chromatography, indicating that it exists as a homodimer (Supplementary Figure S1). DndB was incubated with DNA fragments spanning −460 to +38 bp with respect to the transcription start site of the *dndBCDE* operon. As shown in Fig. 2, the addition of DndB to the reaction mixture caused a specific shift in the mobility of DNA fragment B_1_, extending from position −85 bp to +38 bp. In contrast, no shift was observed for the DNA fragments B_2_ and B_3_, spanning −234 bp to −85 bp and −460 bp to −232 bp, respectively, relative to the transcription start site. The 123-bp DNA fragment B_1_ was PCR-fused to a promoterless *lacZ* gene and cloned into a pEASY-Blunt Zero plasmid, generating pWHU1809. β-Galactosidase activity was compared in wild-type *S. enterica* serovar Cerro 87 and *dptB*^***−***^ strain pWHU1809, revealing that the level of β-galactosidase activity in *dptB*^−^ (pWHU1809) was more than 2-fold higher than that in the wild-type strains (Supplementary Figure S2). Taken together, these results indicate that the 123 bp DNA fragment B_1_ harbors the essential elements for DndB binding.

### DndB binds to two palindromic inverted repeat motifs

**Figure 3:**
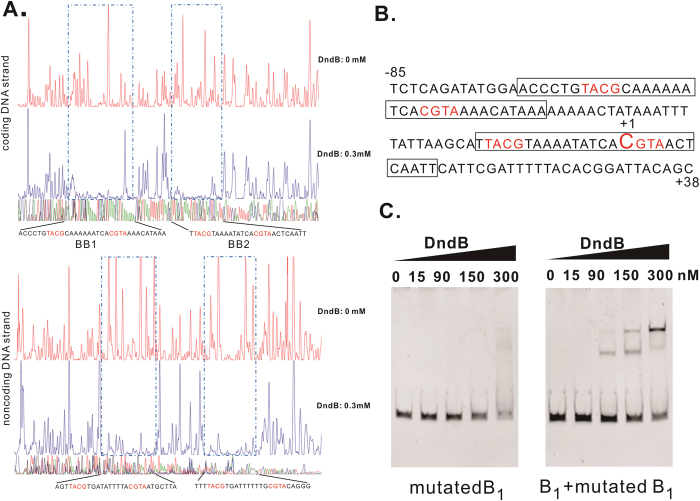
DndB binds to two regions in the promoter of the *dndBCDE* operon. (**A**) Footprinting assays identifying two regions protected by DndB, BB1 and BB2, on both the coding and noncoding DNA strands. The palindromic motifs are shown in red letters. (**B**) The promoter sequence of the *dndBCDE* operon was analyzed using BPROM and Neutral Network Promoter Prediction online software. (**C**) EMSAs were performed on serial concentrations of DndB ranging from 0 to 300 nM with the DNA fragments B_1_ and mutated B_1_. The TACG in BB1 and CGTA in BB2 were replaced by GGTT and TTGG respectively, in the mutated B_1_ fragment. Mutation of the B1 fragment impaired the DNA-binding activity of DndB. Shifted bands were recovered when the wild-type DNA B1 fragment was added.

A DNase I footprinting assay was performed to identify the exact DNA sequences to which DndB binds in DNA fragment B_1_. The assay revealed two protected regions, BB1 and BB2 (Fig. 3A). BB1 covered a protected region of 33 nt extending from nucleotide −72 to −40 on the coding strand and encompassing 26 nt on the complementary strand. The BB2 region spanned nucleotides −66 to −49 and −14 to +4 on the coding and complementary strands, respectively. The footprinting assay further revealed a putative palindromic motif of 5′-TACGN^10^CGTA-3′ that was formed from two inverted repeats separated by ten nucleotides in both the BB1 and BB2 regions (Fig. 3B). The palindromic motifs were found to be essential for DndB binding because the gel mobility assays showed that mutations in TACG in BB1 and CGTA in BB2 regions dramatically decreased the DndB-DNA interaction (Fig. 3C). The mutation of the two 5′-TACGN^10^CGTA-3′ still displayed residual binding to DndB. This result might due to the remaining CGTA/TACG left in the two palindromic motifs.

### Cys29 plays an essential role in regulation

**Figure 4:**
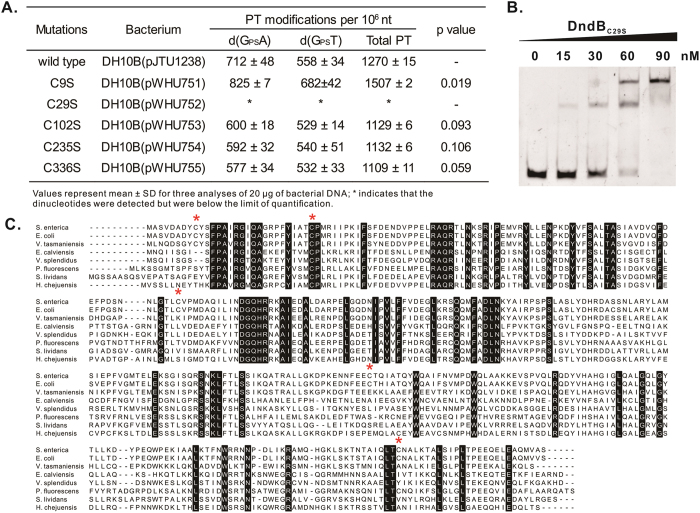
Effect of cysteine mutations on DndB regulation. (**A**) PT frequencies in *E. coli* DH10B harboring pJTU1238 and its derivatives. (**B**) EMSA was performed with serial concentrations of DndB_C29S_ ranging from 0 to 90 nM with the B_1_ fragment. (**C**) Amino acid sequence alignment of DndB homologs (cysteine residues are marked by a red asterisk) from *S. enterica* serovar Cerro 87 (GW13_PRO3447), *E. coli* B7A (AIF62361), *Vibrio tasmaniensis* 1F-267 (WP_017104390), *Enterovibrio calviensis* 1F-230 (WP_017017144), *Vibrio splendidus* ZS-139 (WP_017074154), *Pseudomonas fluorescens* pf0-1 (YP_346470), *Streptomyces lividans* (WP_003972933) and *Hahella chejuensis* KCTC2396 (WP_011400707).

Cysteine residues often play key roles in transcriptional regulation. For example, OxyR utilizes cysteine as a regulatory switch to respond to redox changes in the cellular environment, and the SarA/MgrA family transcriptional regulators employ cysteine residues to mediate bacterial virulence and antibiotic resistance^19^,^20^. To investigate whether the cysteine residues in DndB were important for PT regulation, we replaced each of the five cysteine residues with a serine by site-directed mutagenesis. These replacements were performed in plasmid pJTU1238, which harbored the *dndBCDE* operon of *S. enterica* serovar Cerro 87 and we introduced these PT modifications into *E. coli* DH10B DNA^13^. The PT modifications were quantified to measure regulatory changes in DndB and its derivatives. As shown in Fig. 4A, the C9S, C102S, C235S and C336S mutations had no significant effects on total PT modification frequency. However, the substitution of C29 with a serine dramatically reduced the frequency from 1270 ± 15 per 10^6^ nt to a barely detectable level, suggesting that C29 plays an essential role in the binding or conformation of DndB. The EMSA results showed that DndB_C29S_ displays a stronger binding affinity for the promoter region (Fig. 4B). The DNA fragment B_1_ was completely shifted by only 90 nM DndB_C29S_, although the same shift required 300 nM of native DndB. This finding corresponds well with the sequence alignment, which showed that C29 is a highly conserved residue in native DndB proteins from different bacteria (Fig. 4C).

PT modifications endow DNA with a chemical reducing property and protect host bacteria against peroxides^9^. When bacteria are exposed to hydrogen peroxide, sulfur in the DNA backbone is consumed, and DNA is converted to a PT-free state^9^. PT DNA can thus be regarded as a new type of antioxidant in bacteria. The transcriptional regulator function of DndB prompted us to investigate whether DndB is involved in the oxidative stress response by regulating genomic PT abundance. Here we tested the transcription of the *dnd* operon in response to oxidative stress as well as carbonyl stress, including hydrogen peroxide, hypochlorous acid (HOCl) and formaldehyde. However, no significant changes in the *dnd* operon measured by qRT-PCR were observed upon exposure to these stresses (Table 2).

## Discussion

The DNA PT system consists of two components, modification enzymes (DndABCDE) and restriction cognates (DndFGHI). Members of DndABCDE modify a specific phosphodiester backbone within the GAAC/GTTC recognition sequence, and DndFGHI proteins catalyze the formation of double-stranded breaks in non-PT-protected DNA^6^,^14^,^16^. The DNA PT systems share similarities with methylation-based RM systems and are thus regarded as a new type of bacterial defense system. RM systems are selfish genetic elements that kill cells that have eliminated them, leading to post-segregational cell killing^21^. However, the deletion of *dndBCDE* as well as *dndFGHI* in *S. enterica* serovar Cerro 87 had no effect on cell viability^6^. In addition, GAAC/GTTC sites across genomes were partially modified and a given GAAC/GTTC site was not consistently PT modified in the bacterial population^16^. The unique feature of partial modification densities across the genome in the presence of active restriction enzymes make DNA PT systems different from the known RM systems and suggest the presence of unidentified physiological characteristics.

A total of 53 positive hits (with BLASTN e-value = 0, identity  99% and coverage  96%) were obtained from both databases of non-redundant nucleotide collection (nr/nt) and whole genome shotgun contigs (WGS) with *Salmonella* set as ‘organism of selection’ using the *dndBCDE* operon of *S. enterica* serovar Cerro 87 as the query. All strains are *Salmonella enterica* subsp. enterica with diverse serotypes of Cerro, Saintpaul, Panama, Bareilly, Mbandaka and Namur. The current study in *S. enterica* serovar Cerro 87 revealed that PT modifications are regulated by DndB, which functions as a transcriptional regulator to control genomic PT modifications by influencing the level of DndBCDE expression. PT modifications in *dptB*^***−***^ strictly occurred at d(G_PS_A) and d(G_PS_T) sites at a 1:1 ratio, suggesting that DndB regulates the transcription of the *dnd* operon but plays no role in PT sequence selectivity. DndB bound to two regions of the *dndBCDE* operon, each of which contains a 5′-TACGN^10^CGTA-3′ palindromic box. Mutations of both boxes significantly decreased the binding affinity of DndB. DndB_C29S_ showed a stronger binding affinity for the promoter, leading to a sharp decrease in DNA PT modifications *in vivo*. However, DndB_C29S_ still possessed residual DNA binding and barely detectable PT modifications. These data raise two possibilities, namely, that C29 may be involved in direct binding to the promoter or that C29 may have critical effects on DndB conformation. In the future, the availability of the DndB structure may allow for a better understanding of the interaction between DndB and its DNA substrates, and the mechanism by which the substitution of C29 alters the structure of the DndB/DNA complex could be explored. Bioinformatics pattern searches revealed that the 5′-TACGN^10^CGTA-3′ motif is located in upstream regions, 200 bp prior to the start codon, of eleven genes—GW13_PRO0283, GW13_PRO0295, GW13_PRO0296, GW13_PRO0509, GW13_PRO0798, GW13_PRO2418, GW13_PRO2485, GW13_PRO2826, GW13_PRO3447, GW13_PRO3539 and GW13_PRO4223. Among them, only GW13_PRO3447, encoding DndB protein, harbors two 5′-TACGN^10^CGTA-3′ motifs in the promoter region. The other ten genes were not differentially expressed upon *dndB* deletion.

Xie *et al.* reported that PT modified DNA functions as a peroxide reducing reagent, enabling the wild-type *S. enterica* serovar Cerro 87 to be more resistant to H_2_O_2_ than *dnd* gene mutants^9^. However, qRT-PCR showed no significant changes of *dnd* cluster upon exposure to oxidants of H_2_O_2_ and HOCl. This suggests that DndB does not function as a redox-sensing transcriptional regulator. This study was unable to identify the triggering factors that act as an on/off switch for the PT system through DndB. This goal should be pursued in future studies.

Bacterial defense systems, e.g., RM systems, require the tight regulation of restriction and modification enzymes to provide effective protection. Initially, it is essential that methylation precede endonuclease restriction activity in the host cell to prevent autorestriction. Subsequently, the level of methyltransferase activity is expected to be reduced because excessive methylation may decrease the efficiency of restriction activity against viral DNA or lead to DNA mutations^22^,^23^. Proteomic profiling identified a group of proteins in the replication COG group, including UvrA, MutS, DNA gyrase subunit A, topoisomerase IV subunit B, and DNA polymerase I, that were up-regulated in *dptB*^***−***^. The induction of the SOS response (UvrA) suggests that phosphorothioation possibly affect DNA replication. DNA gyrase and topoisomerase IV are type II bacterial DNA topoisomerases that are essential for the maintenance of DNA topological homeostasis and for solving topological problems linked to DNA replication and transcription^24^. UvrA and MutS participate in NER and MMR, respectively. In *E. coli*, NER is carried out by the UvrABC complex to repair lesions caused by exogenous damage^25^. The MMR system recognizes and removes single-base mismatches and small nucleotide insertions or deletions that result from errors introduced during replication^26^. NER and MMR are closely linked and represent two primary pathways for the removal of DNA damage to maintain genomic stability. Although sulfur replacement is a minor modification compared to other DNA changes, an *in vitro* excision assay with UvrABC revealed that the PT-modified DNA backbone is recognized as a substrate by NER, albeit at a low efficiency^27^. The disruption of *dndB* resulted in 18.8-, 9.5- and 15.4- fold increases in DndC, DndD, and DndE, respectively. In contrast, the total PT sites in *dptB*^***−***^ only increased by 2-fold, accounting for 16% of 32,795 genomic GAAC/GTTC sites. Proteomic analysis implied that genomic GAAC/GTTC sites might have a limitation that prevents PT from being modified; otherwise, excess PT would be regarded as abnormal, inducing a massive DNA repair response. The most marked changes in protein abundance involved the *dnd* operon itself, the only other proteins showing at least a 5-fold change are involved in DNA repair (UvrA) or envelope stress (RcsD). These observations appear to be consistent with the idea that *dnd* is a selfish genetic element.

This study provides insights into the regulatory mechanisms underlying DNA PT modifications. DndB binds to two palindromic inverted repeats in the promoter region. The control of PT modification might be a consequence of an unknown environmental or cellular stimulus of DndB, acting as a switch to regulate modification frequency. Proteomic analysis identified global changes in response to *dndB* deletion and increased PT modifications, shedding light on PT biology. *dndB* is co-transcribed as a single operon with *dndCDE*, and *dndBCDE* operons are widely present in prokaryotes and have a conserved genetic organization^2^,^28^. The aggravated DNA degradation phenomenon was also observed in *dndB*-deficient mutant of *S. lividans*^18^. These findings imply that transcriptional regulation of the *dnd* operon by DndB might be widely employed in DNA PT systems.

## Materials and Methods

### Bacterial strains, plasmids and primers

All strains and plasmids used in this work are listed in Table 3. Primers are listed in Supplementary Table S1. *Salmonella* and *E. coli* strains were grown in Luria-Bertani (LB) broth. For *dptB*^***−***^ complementation, pWHU746 was constructed by PCR amplifying the DNA fragment containing the *dndB* gene and the upstream promoter region using dndB-F and dndB-R as primers. The 1299 bp fragment was inserted into plasmid pBluescript II SK(+) to yield pWHU746.

### Protein expression and purification

The *dndB* gene was PCR-amplified using the *S. enterica* serovar Cerro 87 chromosome as a template and cloned into *EcoR*I and *Xho*I digested pSJ7, a pET43a derivative, to yield pJTU3522 (a generous gift from Dr. Jingdan Liang). pJTU3522 was transformed into *E. coli* BL21 (DE3) and DndB protein was expressed as a N-terminal NusA-tagged protein containing a poly-His tag and the tobacco etch virus (TEV) recognition site. Cells were grown at 37 °C in Luria broth medium to an A_600_ of 0.6-0.8 and then induced overnight at 16 °C with 0.5 mM IPTG. The cells were harvested by centrifugation at 6000 × g for 10 min at 4 °C and resuspended in buffer A (50 mM Tris-HCl, pH 8.0, 300 mM NaCl, and 40 mM imidazole). The resuspended cells were lysed by sonication and centrifuged at 14,000 × g for 30 min. The supernatant was then applied to a 5 mL HisTrap^TM^ HP chelating column (GE Healthcare). The NusA-tag and His-tag of the purified DndB protein were removed by TEV protease in phosphate-buffered saline (PBS), and the protein concentration was determined using the Bradford method^29^. The native molecular mass of DndB was estimated by size exclusion chromatography using a HiLoad^TM^ 16/60 Superdex^TM^ 200 column (GE Healthcare). The column was equilibrated with 20 mM Tris-HCl, pH 8.0, and 150 mM NaCl, and elution was performed with the same buffer at a flow rate of 0.7 mL/min. Calibration was performed with myoglobin (17 kDa) ovalbumin (44 kDa), albumin (66 kDa), phosphorylase b (97 kDa), and γ-globulin (158 kDa).

### Quantification of PT modifications

The LC-MS/MS method used for PT quantification has been described previously^13^. Briefly, DNA hydrolytes were resolved using a Thermo Hypersil GOLD aQ column (150 × 2.1 mm, 3 μm). Elution was performed at 35 °C, beginning with incubation in 97% buffer A (0.1% acetic acid in water) and 3% buffer B (0.1% acetic acid in acetonitrile) for 5 min followed by an increase in buffer B from 3% to 98% over another 30 min at a flow rate of 0.3 mL/min. The LC column was coupled to a Thermo TSQ Quantum Access MAX mass spectrometer with an electrospray ionization source in positive mode. The multiple reaction monitoring (MRM) mode was employed for the detection of daughter ions derived from precursor ions. All instrument parameters were optimized for maximal sensitivity^13^.

### Quantitative real-time PCR

To monitor DndB regulation in response to diverse environmental stresses, cell cultures with an A_600_ of 0.8 were treated with 1 mM H_2_O_2_, 0.4 mM HOCl and 1 mM formaldehyde for 10 min, followed by RNA extraction. Total RNA was isolated using an RNeasy Protect Bacteria Mini Kit (Qiagen) followed by DNase I treatment. The RNA was reverse transcribed using a RevertAid First Strand cDNA Synthesis Kit (Thermo Scientific). Real-time RT-PCR was performed using a SsoFast EvaGreen Supermix with Low ROX Kit (Bio-Rad) and a 7900HT Fast Real-Time PCR System (Applied Biosystems). To quantitatively compare transcription of the *dnd* operon, the primers dndB-RT-1 and dndB-RT-2 were designed according to the *dndCD* genes (Supplementary Table S1). The housekeeping gene *gapA*, which encodes GAPDH, was used for normalization. mRNA levels were analyzed using the comparative threshold cycle (2^−ΔΔCt^) method.

### Site-directed mutagenesis of *dndB*

Site-directed mutagenesis of the *dndB* gene in plasmid pJTU1238, which harbored the *dndBCDE* cluster of *S. enterica* serovar Cerro 87, was conducted using a Muta-direct Site Directed Mutagenesis Kit (SBS Genetech). The five cysteine residues were individually replaced with serine. The primers were designed according to the manufacturer’s recommendations (Table S1). Mutations in pJTU1238 were verified by DNA sequencing. The derived plasmids were transformed into *E. coli* DH10B to detect PT modifications by LC-MS/MS.

### Electrophoretic mobility shift assay (EMSA)

Putative DNA promoter regions were PCR-amplified or commercially synthesized, purified with an Omega Gel Extraction Kit and dissolved in water. The purified DndB protein or mutated derivatives were incubated with 20 nM DNA fragments in 20 μL of binding buffer (20 mM Tris-HCl, pH 8.0, 50 mM KCl, 10 mM MgCl_2_, 5 mM EDTA, 10 mM DTT, and 5% glycerol). The reaction mixtures were incubated at room temperature for 30 min and then loaded onto 6% native polyacrylamide gels. Electrophoresis was performed using 0.5 × TBE buffer (44.5 mM Tris, 44.5 mM boric acid, and 1 mM EDTA) at room temperature. The gel was stained with GelRed and photographed.

### β-Galactosidase assays

To analyze the binding of DndB to DNA fragment B1, plasmid pWHU1809 was constructed for the following β-galactosidase assay. First, the 123 bp B1 fragment and promoterless *lacZ* gene were generated by PCR using the primer pairs OverB-LL/OverB-LR and OverB-RL/OverB-RR, respectively (Supplementary Table S1). OverB-LR and OverB-RL are chimeric primers. A mixture of two purified PCR products, which overlapped by 36 bp, served as the template for a ligation PCR using primers overB-LL and overB-RR. A 3,294 bp product was obtained and cloned into the pEASY-Blunt Zero vector (TransGen Biotech), yielding plasmid pWHU1809. β-Galactosidase activity was determined in Miller units using the protocol described by Zhang *et al.*^30^,^31^. Four 20-μL samples of culture were obtained at an A_600_ of between 0.2 and 0.6 and mixed with 80 μL of permeabilization solution (100 mM Na_2_HPO_4_, 20 mM KCl, 2 mM MgSO_4_, 0.8 mg/mL CTAB, 0.4 mg/mL sodium deoxycholate and 5.4 μL/mL β-mercaptoethanol). The permeabilization mixture was incubated at 30 °C for approximately 30 min, and 600 μL of substrate solution (60 mM Na_2_HPO_4_, 40 mM NaH_2_PO_4_, 1 g/mL *o*-nitrophenyl-β-D-galactoside and 2.7 μL/mL β-mercaptoethanol) prewarmed to 30 °C was added to initiate the reaction. After 0.5-2 h at 30 °C, the reactions were terminated by the addition of 700 μL of 1 M Na_2_CO_3._ The rate of change in A_420_/hour per A_600_ unit of culture was then converted into Miller units.

### Dye primer-based DNase I footprinting assay

DNase I footprinting assays were performed using FAM-labeled primers^32^. For preparation of the sense-strand probe, the promoter region of DndB was PCR-amplified with Dpx DNA polymerase (TOLO Biotech) using the primers B_1_-F and B_1_-R (Table S1). The amplicon was then purified and further cloned into a HincII-digested pUC18H vector (TOLO Biotech). The obtained plasmid was verified by DNA sequencing and used as a template for the further preparation of fluorescent FAM-labeled probes with the primers M13F-47(FAM)/M13R-48 and M13R-48(FAM)/M13F-47^32^. The FAM-labeled probes were purified with a Wizard^®^ SV Gel kit and a PCR Clean-Up System (Promega) and quantified with a NanoDrop 2000C (Thermo). For each assay, 200 ng of probes were incubated with different amounts of DndB in a total volume of 40 μL. After incubation for 30 min at 25 °C, 10 μL of a solution containing approximately 0.015 units of DNase I (Promega) and 100 nmol of freshly prepared CaCl_2_ was added and further incubated for 1 min at 25 °C. The reaction was stopped by adding 140 μL of DNase I stop solution (200 mM unbuffered sodium acetate, 30 mM EDTA and 0.15% SDS). The samples were first extracted with phenol/chloroform and then precipitated with ethanol, and the pellets were dissolved in 30 μL of MilliQ water. The preparation of the DNA ladder, electrophoresis and data analysis were performed as described previously by Wang *et al.*^32^, with the exception that the GeneScan-LIZ500 size standard (Applied Biosystems) was used.

### Proteomic analysis

Wild-type *S. enterica* and *dndB* mutant strains were grown in LB medium at 30 °C to an A_600_ of 0.8. The bacteria were centrifuged at 10,000 × g for 2 min, mixed with Laemmli sample buffer and heated at 95 °C for 5 min. The denatured proteins were separated by 10% SDS-PAGE, and electrophoresis was stopped when the bromophenol blue band reached ~1 cm below the stacking gel. Sample preparation and in-gel digestion were performed as described previously by Hu *et al.*^33^. The digested peptides were extracted by incubating the gels twice with 50% ACN and 5% formic acid for 20 min at 37 °C. The extracted peptides were vacuum-dried and dissolved in HPLC-grade water prior to LC-MS/MS analysis.

The peptides were resolved using a reverse-phase capillary column (75 μm × 15 cm) packed in-house with 100 Å, 5 μm Magic C18AQ silica-based particles (Michrom BioResources). Elution was initiated with 93% buffer A (97% H_2_O, 3% ACN and 0.1% formic acid) and 7% buffer B (100% ACN and 0.1% formic acid) for 3 min, and then buffer B was increased to 28% over another 60 min at a flow rate of 300 nL/min. The peptides eluted from the capillary column were electrosprayed directly onto an LTQ Velos_Pro mass spectrometer (Thermo Scientific) for MS and MS/MS analyses. The LC-MS/MS data were analyzed using Mascot software (version 2.3.02) to perform a search against the *S. enterica* protein database. Cysteine carbamidomethylation and methionine oxidation were chosen as the fixed modification and variable modification, respectively. The precursor mass error tolerance was set to 1.5 Da, and the fragment mass error tolerance was set to 0.8 Da. The maximum number of missed cleaved sites allowed was 2. False discovery rates (FDRs) were allowed at below 1% for the peptide and protein identifications. Three biological replicates were used for proteomic analysis of wild-type *S. enterica* and *dptBˉ*. Proteins with an average fold change of >1.5 or <0.66 were considered up- and down-regulated, respectively (*p*-value < 0.05, Student’s *t*-test).

## Additional Information

**How to cite this article**: He, W. *et al.* Regulation of DNA phosphorothioate modification in Salmonella *enterica* by DndB. *Sci. Rep.* **5**, 12368; doi: 10.1038/srep12368 (2015).

## Supplementary Material

Supplementary Information

## Figures and Tables

**Table 1:** Comparison of total PT in wild-type *S. enterica* serovar Cerro 87 and *dptB*^−^.

	**PT modifications per 10^6^ nt**			
**Bacterium**	**d(G_PS_A)**	**d(G_PS_T)**	**Total PT**	***p* value**
*S. enterica* serovar Cerro 87	297 ± 4	297 ± 7	594 ± 11	—
*dptB*ˉ	577 ± 95	581 ± 97	1158 ± 192	0.036
*dptB*ˉ(pWHU746)	327 ± 10	305 ± 2	632 ± 12	0.023

Values represent the means ±SD for three analyses of 20 μg of bacterial DNA. *dptB*^*−*^(pWHU746) is the *dptB*^−^ mutant complemented with pWHU746, containing *dndB* with the promoter. *p* values were determined using a two-sided Student’s test for samples compared to wild-type *S. enterica* serovar Cerro 87.

**Table 2:** Quantification of *dnd* gene transcription by quantitative real-time PCR.

	**Average C_T_ ± SD**		
**Bacterium**	***dndCD***	***gapA***	*dndCD* fold change relative togapA*
*S. enterica*	22.00 ± 0.21	17.76 ± 0.39	1 (0.8–1.1)
*dptB*^*−*^	17.75 ± 0.19	17.43 ± 0.28	15.24 (14.22–16.34)
*dptB*^*−*^(pWHU746)	26.74 ± 0.14	25.63 ± 0.27	0.32 (0.1–1.0)
*S. enterica* (H_2_O_2_, 1mM)	24.84 ± 0.05	23.22 ± 0.18	0.32 (0.27–0.39)
*S. enterica* (HOCl, 0.4mM)	21.77 ± 0.21	23.67 ± 0.15	0.97 (0.66–1.42)
*S. enterica* (formaldehyde, 1mM)	21.56 ± 0.38	22.64 ± 0.45	0.77 (0.35–1.71)

Values represent the means ± SD for three analyses.

^*^The transcriptional changes of *dndCD* relative to the housekeeping gene, *gapA.* Quantitative real-time PCR data analysis was performed according to the comparative threshold cycle (C_T_) method, also known as 2^−ΔΔC^_T_.

**Table 3:** Bacterial strains and plasmids.

**Strains or plasmids**	**Relevant properties**	**Source or reference**
**Strains**		
*S. enterica* serovar Cerro 87	strain naturally contains the *dndBCDE* gene cluster and PT-modified G_PS_A and G_PS_T	7
*dptB*ˉ	*S. enterica d*erivative, *dndB* in-frame deletion mutant	9
XTG102	*S. enterica d*erivative, *dndB-E* in-frame deletion mutant	7
**Plasmids**		
pBluescript II SK( + )	cloning vector, Amp^r^	34
pSJ7	cloning vector, Amp^r^	35
pJTU3522	*dndB* from *S. enterica*, cloned in pSJ7	Gift from Jingdan Liang
pZWHJ002	*lacZ* promoterless, derivative of pXMJ19, Cam^r^	36
pJTU1238	*dndBCDE* from *S. enterica*, cloned in SK(+)	7
pWHU1809	B_1_ fragment with promoterless *lacZ* cloned in pEASY-Blunt Zero (TransGen Biotech)	This study
pWHU746	*dndB* with promoter from *S. enterica*, cloned in SK(+)	This study
pWHU751	pJTU1238 derivative site mutant with C9S	This study
pWHU752	pJTU1238 derivative site mutant with C29S	This study
pWHU753	pJTU1238 derivative site mutant with C102S	This study
pWHU754	pJTU1238 derivative site mutant with C235S	This study
pWHU755	pJTU1238 derivative site mutant with C336S	This study
