# Supplementary material for: Regulation of DNA phosphorothioate modification in Salmonella enterica by DndB
Source: Sci Rep. 2015 Jul 20;5:12368. doi: 10.1038/srep12368 (PMC4507180; doi:10.1038/srep12368)
Supplement: Supplementary Information [file srep12368-s1.doc]

**Supplementary Information for “Regulation of DNA phosphorothioate modification in *Salmonella enterica* by DndB”**

Wei He^1,#^, Teng Huang^1,2,#^, You Tang^1^, Yanhua Liu^3^, Xiaolin Wu^1^, Si Chen^1^, Wan Chan^4^, Yajie Wang^1,2^, Xiaoyun Liu^3^, Shi Chen^1*^, Lianrong Wang^1*^

*^1^ Key Laboratory of Combinatorial Biosynthesis and Drug Discovery, Ministry of Education, and School of Pharmaceutical Sciences, Wuhan University, Wuhan 430071, China*

*^2^ Taihe Hospital, Hubei University of Medicine, Shiyan, Hubei, China*

*^3^ Institute of Analytical Chemistry and Synthetic and Functional Biomolecules Center, College of Chemistry and Molecular Engineering, Peking University, Beijing 100871, China*

*^4^ Department of Chemistry, The Hong Kong University of Science and Technology, Clear Water Bay, Kowloon, Hong Kong, China*

**Table S1. Primers**

| **Primers** | **Sequence** | **Source or reference** |
| --- | --- | --- |
|  |  |  |
| **Electrophoresis mobility shift assays** | |  |
| B_1_-F | TCTCAGATATGGAACCCT | This study |
| B_1_-R | GCTGTAATCCGTGTAAAA | This study |
| B_2_-F | TGCCTGGAGTTAATTTGG | This study |
| B_2_-R | AGTGCTCGTGGCTGCTGTG | This study |
| B_3_-F | ACAGGGTAACCACGCAGCA | This study |
| B_3_-R | GCATCTGAGCCGTTTGGC | This study |
| **Quantitative RT-PCR** | |  |
| dndB-RT-1 | CAGGACGAAGTTGAAAAGC | This study |
| dndB-RT-2 | ACTATCGGGCGAGGGTTGA | This study |
| GAPDH-RT-1 | TTATCGTACCAGGAGACCAG | This study |
| GAPDH-RT-2 | CGAAGGCGAAATGAAAGG | This study |
| **Site-directed mutagenesis** | | |
| C9S-F | TTGATGCAGACTAC**TCC**TACTCGTTTCCGGC | This study |
| C9S-R | GCCGGAAACGAGTA**GGA**GTAGTCTGCATCAA | This study |
| C29S-F | ATATTGCCACA**TCC**CCGA | This study |
| C29S-R | TCGG**GGA**TGTGGCAATAT | This study |
| C102S-F | ACCTGGGGACGTTA**TCC**GTGCCAATGGATGC | This study |
| C102S-R | GCATCCATTGGCAC**GGA**TAACGTCCCCAGGT | This study |
| C235S-F | GAACAACTTTGAGGAA**TCC**ACCCAAATAGCTACCC | This study |
| C235S-R | GGGTAGCTATTTGGGT**GGA**TTCCTCAAAGTTGTTC | This study |
| C336S-F | GCGATCCAGCTCACC**TCT**AATGCGCTAAAAACC | This study |
| C336S-R | GGTTTTTAGCGCATT**AGA**GGTGAGCTGGATCGC | This study |
| **Plasmid construction** |  |  |
| OverB1-LL | GGATCCTCTCAGATATGGAACCCT | This study |
| OverB1-LR | ATCCGTAATCATGGTCATGCTGTAATCCGTGTAAAA | This study |
| O verB1-RL | TTTTACACGGATTACAGCATGACCATGATTACGGAT | This study |
| OverB1-RR | AAGCTTTACGCTGCCAGCTCATAG | This study |
| dndB-F | TCTAGAGTATCAGCGTCATTCTCCC | This study |
| dndB-R | AAGCTTGTATTCGGCCAAATCGTAG | This study |

**Table S2. Differentially expressed proteins in *dptBˉ* and XTG102 compared with wild-type *S. enterica* serovar Cerro 87**

|  |  | | **Fold change^a^** | |  |
| --- | --- | --- | --- | --- | --- |
| **COG Classification** | | **genbank ID** | **dptBˉ** | **XTG102** | **Function** |
| C | GW13_PRO2474 | | **2.33** | **3.67** | Phosphoribulokinase homolog, function unknown |
| C | GW13_PRO4021 | | **0.66** | **2.00** | Cytochrome O ubiquinol oxidase subunit II |
| C | GW13_PRO2156 | | **0.33** | 1.89 | Uptake hydrogenase small subunit precursor |
| C | GW13_PRO2009 | | **0.59** | 1.78 | Oxidoreductase, aldo/keto reductase family |
| C | GW13_PRO2486 | | **1.68** | 1.50 | Nitrite reductase [NAD(P)H] large subunit |
| C | GW13_PRO0095 | | **3.33** | 1.30 | trifunctional transcriptional regulator/proline dehydrogenase/pyrroline-5-carboxylate dehydrogenase |
| C | GW13_PRO0535 | | **4.00** | 1.29 | Pyruvate-flavodoxin oxidoreductase |
| C | GW13_PRO4325 | | **1.50** | 1.27 | 2-oxoglutarate dehydrogenase E1 component |
| C | GW13_PRO3100 | | **1.75** | 1.04 | Phosphoenolpyruvate carboxylase |
| C | GW13_PRO3716 | | **1.55** | 0.99 | Aconitate hydratase 2 |
| C | GW13_PRO3151 | | **0.66** | 0.68 | Malate synthase |
| C | GW13_PRO3998 | | **0.53** | **0.56** | Putative oxidoreductase |
| C | GW13_PRO0470 | | **1.71** | **0.54** | Aconitate hydratase; 2-methylisocitrate dehydratase |
| C & H | GW13_PRO2977 | | **0.42** | 0.94 | Protoporphyrinogen IX oxidase, oxygen-independent, HemG |
| C & H & R | GW13_PRO0539 | | 1.04 | **1.65** | D-lactate dehydrogenase |
| C & H & R | GW13_PRO2653 | | **0.52** | 1.12 | 2-ketoaldonate reductase, broad specificity |
| D | GW13_PRO3685 | | 0.77 | **2.11** | Cell division protein FtsA |
| E | GW13_PRO3171 | | 0.61 | **3.67** | Aspartokinase |
| E | GW13_PRO1783 | | 2.50 | **3.06** | L-proline glycine betaine binding ABC transporter protein ProX |
| E | GW13_PRO1325 | | **2.50** | 3.03 | ABC transporter, periplasmic substrate-binding protein |
| E | GW13_PRO0452 | | 2.67 | **2.83** | Tryptophan synthase alpha chain |
| E | GW13_PRO0373 | | 0.69 | **2.67** | D-amino acid dehydrogenase small subunit |
| E | GW13_PRO0989 | | 1.06 | **2.17** | Putrescine transport ATP-binding protein PotA |
| E | GW13_PRO1185 | | 0.92 | **1.94** | Histidinol dehydrogenase |
| E | GW13_PRO0680 | | **2.89** | 2.15 | Dipeptidyl carboxypeptidase Dcp |
| E | GW13_PRO3898 | | 1.18 | **1.77** | Gamma-glutamyl phosphate reductase |
| E | GW13_PRO2671 | | **0.27** | 1.83 | Valine--pyruvate aminotransferase |
| E | GW13_PRO2939 | | **3.00** | 1.83 | Diaminopimelate epimerase |
| E | GW13_PRO4315 | | 0.83 | **1.61** | Allophanate hydrolase 2 subunit 1 |
| E | GW13_PRO4460 | | **0.56** | 1.33 | Putrescine ABC transporter putrescine-binding protein PotF |
| E | GW13_PRO1058 | | **0.61** | 1.30 | N-methyl-L-amino-acid oxidase ; N-methyl-L-tryptophan oxidase |
| E | GW13_PRO2058 | | **1.70** | 1.28 | Glycine dehydrogenase |
| E | GW13_PRO0702 | | **0.61** | 1.22 | L-proline glycine betaine ABC transport system permease protein ProV |
| E | GW13_PRO0939 | | **0.63** | 1.18 | Nickel ABC transporter, periplasmic nickel-binding protein nikA2 |
| E | GW13_PRO3157 | | **2.33** | 1.06 | 5-methyltetrahydrofolate--homocysteine methyltransferase |
| E | GW13_PRO4415 | | 1.06 | **0.50** | Glutamate transport membrane-spanning protein |
| E | GW13_PRO4377 | | 0.61 | **0.44** | Urocanate hydratase |
| E & H | GW13_PRO3446 | | **18.78** | **0.10** | 3'-phosphoadenosine 5'-phosphosulfate sulfurtransferase DndC |
| E & M | GW13_PRO2350 | | 0.98 | **8.60** | N-acetylneuraminate lyase |
| E & T | GW13_PRO4268 | | 1.07 | **0.55** | Glutamate Aspartate periplasmic binding protein precursor GltI |
| E & T | GW13_PRO1446 | | 0.56 | **0.48** | Lysine-arginine-ornithine-binding periplasmic protein precursor |
| F | GW13_PRO4264 | | 0.91 | **3.00** | Inosine-uridine preferring nucleoside hydrolase |
| F | GW13_PRO1660 | | **4.00** | 0.62 | Phosphoribosylformylglycinamidine synthase, synthetase subunit/Phosphoribosylformylglycinamidine synthase, glutamine amidotransferase subunit |
| G | GW13_PRO2788 | | 1.06 | **3.33** | Hexose phosphate transport protein UhpT |
| G | GW13_PRO2348 | | 0.88 | **3.17** | N-acetylmannosamine-6-phosphate 2-epimerase |
| G | GW13_PRO4072 | | 0.97 | **2.44** | Inosine-guanosine kinase |
| G | GW13_PRO2275 | | **4.33** | **2.22** | Phosphoheptose isomerase |
| G | GW13_PRO2533 | | **1.74** | 2.03 | Glycogen phosphorylase |
| G | GW13_PRO3040 | | 1.48 | **1.78** | Rhamnulokinase |
| G | GW13_PRO2887 | | **1.77** | 1.14 | ATP-binding protein RbsA |
| G | GW13_PRO2526 | | **1.54** | 0.92 | 4-alpha-glucanotransferase (amylomaltase) |
| G | GW13_PRO2666 | | **0.33** | 0.78 | Xylose isomerase |
| G | GW13_PRO3940 | | **0.31** | 0.75 | Methylisocitrate lyase |
| G | GW13_PRO3732 | | 3.00 | **0.58** | Polysaccharide deacetylase |
| G & H & R | GW13_PRO1498 | | 1.33 | **0.32** | Pyruvate decarboxylase ; Alpha-keto-acid decarboxylase |
| G & K | GW13_PRO3164 | | **3.33** | 1.89 | Transcriptional regulator |
| G & K | GW13_PRO4279 | | **0.28** | 1.83 | N-acetylglucosamine-6P-responsive transcriptional repressor NagC, ROK family |
| H | GW13_PRO3720 | | 0.70 | **7.33** | 4-hydroxythreonine-4-phosphate dehydrogenase |
| H | GW13_PRO1397 | | 0.94 | **5.33** | O-succinylbenzoate synthase |
| H | GW13_PRO3136 | | 1.46 | **3.42** | Uroporphyrinogen III decarboxylase |
| H | GW13_PRO3115 | | 0.92 | **2.89** | Pantothenate kinase |
| H | GW13_PRO4435 | | 1.00 | **1.66** | Molybdopterin biosynthesis protein MoeA |
| H | GW13_PRO1400 | | 0.89 | **0.32** | 2-succinyl-5-enolpyruvyl-6-hydroxy-3-cyclohexene-1-carboxylic-acid synthase |
| I | GW13_PRO0544 | | 1.33 | **2.67** | FMN-dependent NADH-azoreductase |
| I | GW13_PRO3274 | | 2.17 | **2.67** | Nonspecific acid phosphatase precursor |
| J | GW13_PRO3545 | | 1.17 | **3.00** | tRNA:Cm32/Um32 methyltransferase |
| J | GW13_PRO2291 | | 0.83 | **2.83** | tRNA pseudouridine synthase B |
| J | GW13_PRO3315 | | 1.26 | **1.67** | Translation elongation factor P Lys34:lysine transferase |
| J | GW13_PRO2394 | | 1.48 | **1.65** | Ribosomal protein L11 methyltransferase |
| J | GW13_PRO1956 | | **0.31** | 1.83 | 23S rRNA (Uracil-5-) -methyltransferase RumA |
| J | GW13_PRO2211 | | ^b^n.a | **1.78** | tRNA nucleotidyltransferase |
| J | GW13_PRO0577 | | **0.44** | 1.33 | Ribosomal-protein-L7p-serine acetyltransferase |
| J | GW13_PRO2293 | | **2.12** | 1.05 | Translation initiation factor 2 |
| J | GW13_PRO3427 | | **1.91** | 0.95 | Valyl-tRNA synthetase |
| J | GW13_PRO4251 | | **1.54** | 0.94 | Leucyl-tRNA synthetase |
| J & K & L | GW13_PRO2287 | | **1.54** | 1.11 | Cold-shock DEAD-box protein A |
| K | GW13_PRO2369 | | 0.96 | **2.00** | Transcriptional regulator, GntR family |
| K | GW13_PRO2968 | | 2.47 | **2.33** | Transcriptional activator RfaH |
| K | GW13_PRO0093 | | 0.83 | **1.83** | Transcriptional regulator RutR of pyrimidine catabolism (TetR family) |
| K | GW13_PRO3105 | | 1.13 | **1.73** | Hydrogen peroxide-inducible genes activator |
| K | GW13_PRO2351 | | **2.50** | 1.50 | Transcriptional regulator NanR |
| K | GW13_PRO2606 | | **3.33** | 1.50 | Transcriptional regulator, GntR family |
| K | GW13_PRO2516 | | **1.57** | 1.22 | Transcription accessory protein (S1 RNA-binding domain) |
| K | GW13_PRO2528 | | **3.61** | 0.96 | Transcriptional activator of maltose regulon, MalT |
| K & L | GW13_PRO3646 | | **3.36** | 1.13 | RNA polymerase associated protein RapA |
| K & T | GW13_PRO2829 | | **2.67** | 0.67 | TorCAD operon transcriptional regulatory protein TorR |
| L | GW13_PRO1722 | | 1.33 | **12.33** | DNA repair protein RecN |
| L | GW13_PRO0284 | | 2.00 | **10.33** | Holliday junction DNA helicase RuvB |
| L | GW13_PRO1171 | | 1.33 | **9.22** | DNA gyrase inhibitory protein |
| L | GW13_PRO2943 | | 1.73 | **5.06** | ATP-dependent DNA helicase UvrD/PcrA |
| L | GW13_PRO0283 | | 1.39 | **4.17** | Holliday junction DNA helicase RuvA |
| L | GW13_PRO1735 | | 0.75 | **3.47** | DNA-cytosine methyltransferase |
| L | GW13_PRO3206 | | **5.56** | 3.69 | Excinuclease ABC subunit A |
| L | GW13_PRO1799 | | 0.93 | **2.76** | RecA protein |
| L | GW13_PRO3198 | | 0.63 | **2.85** | Replicative DNA helicase |
| L | GW13_PRO1884 | | **2.13** | 1.36 | DNA mismatch repair protein MutS |
| L | GW13_PRO2988 | | **3.23** | 1.06 | DNA polymerase I |
| L | GW13_PRO2187 | | **1.61** | 1.05 | Topoisomerase IV subunit B |
| L | GW13_PRO1367 | | **2.23** | 0.99 | DNA gyrase subunit A |
| L | GW13_PRO0525 | | 0.83 | **0.58** | Methylated-DNA--protein-cysteine methyltransferase |
| L | GW13_PRO3445 | | **9.51** | **0.03** | DNA sulfur modification protein DndD |
| L & R | GW13_PRO2505 | | 1.18 | **1.94** | ADP compounds hydrolase NudE |
| M | GW13_PRO1808 | | 2.03 | **4.33** | Glucitol operon GutQ protein |
| M | GW13_PRO1064 | | 0.83 | **4.00** | Lipid A biosynthesis lauroyl acyltransferase |
| M | GW13_PRO4339 | | 1.33 | **3.00** | TolA protein |
| M | GW13_PRO1801 | | **0.64** | 2.97 | Membrane-bound lytic murein transglycosylase B precursor |
| M | GW13_PRO3853 | | 1.89 | **2.78** | ClpB protein |
| M | GW13_PRO3326 | | 0.92 | **2.67** | N-acetylmuramoyl-L-alanine amidase |
| M | GW13_PRO3750 | | **1.79** | 1.32 | Multimodular transpeptidase-transglycosylase |
| M | GW13_PRO0328 | | **1.93** | 1.25 | Tail-specific protease precursor |
| M | GW13_PRO1227 | | 2.83 | **0.44** | AsmA protein |
| M | GW13_PRO0848 | | 2.00 | **0.38** | Probable lipoprotein nlpC precursor |
| N | GW13_PRO0133 | | 1.00 | **7.33** | Flagellar motor switch protein FliM |
| N | GW13_PRO0139 | | **0.47** | 3.39 | Flagellar motor switch protein FliG |
| N | GW13_PRO1036 | | 0.56 | **1.89** | Flagellar L-ring protein FlgH |
| N | GW13_PRO0131 | | **0.45** | 1.00 | Flagellar biosynthesis protein FliQ |
| N & T | GW13_PRO3489 | | 0.68 | **1.95** | Methyl-accepting chemotaxis protein I (serine chemoreceptor protein) |
| N & T | GW13_PRO1405 | | **0.31** | 1.67 | Chemotaxis protein CheV |
| N & U | GW13_PRO3538 | | **2.33** | 1.14 | type 1 fimbriae anchoring protein FimD |
| O | GW13_PRO1233 | | 1.00 | **5.39** | Putative heat shock protein YegD |
| O | GW13_PRO3524 | | 2.31 | **5.06** | DNA repair protein RadA |
| O | GW13_PRO3064 | | n.a | **2.67** | hypothetical protein |
| O | GW13_PRO4514 | | **0.47** | 1.59 | Pyruvate formate-lyase activating enzyme |
| P | GW13_PRO4368 | | 1.61 | **8.33** | Molybdenum ABC transporter, periplasmic molybdenum-binding protein ModA |
| P | GW13_PRO0566 | | 0.79 | **1.59** | Glucans biosynthesis protein D precursor |
| P | GW13_PRO3193 | | **2.33** | 0.75 | Zinc uptake regulation protein ZUR |
| P | GW13_PRO1583 | | 1.19 | **0.52** | Arsenate reductase |
| R | GW13_PRO3820 | | 1.10 | **4.83** | Hydroxyacylglutathione hydrolase |
| R | GW13_PRO2228 | | 1.42 | **4.00** | Predicted metal-dependent hydrolase |
| R | GW13_PRO1675 | | 0.97 | **2.48** | GTP-binding protein Era |
| R | GW13_PRO3345 | | 0.90 | **2.33** | YjfP protein |
| R | GW13_PRO3221 | | **2.50** | 2.00 | Xanthine/uracil permease family protein |
| R | GW13_PRO3486 | | 1.78 | **1.73** | Putative GTPases (G3E family) |
| R | GW13_PRO1624 | | **3.33** | 1.31 | Alpha-2-macroglobulin |
| R | GW13_PRO1455 | | **1.83** | 0.54 | Colicin V production protein |
| R | GW13_PRO1403 | | 0.78 | **0.44** | ElaA protein |
| R | GW13_PRO0647 | | n.a | **0.42** | hypothetical protein |
| S | GW13_PRO0296 | | 1.17 | **18.00** | DNA damage-inducible protein YebG,DNA damage-inducible gene in SOS regulon |
| S | GW13_PRO2960 | | 1.33 | **5.06** | DNA recombination protein RmuC |
| S | GW13_PRO0592 | | 0.83 | **2.33** | Putative virulence factor |
| S | GW13_PRO2209 | | 1.20 | **1.98** | Adenylate cyclase |
| S | GW13_PRO3845 | | 2.33 | **2.33** | IcmF like protein |
| S | GW13_PRO2962 | | 1.38 | **1.89** | Protein YigP clustered with ubiquinone biosynthetic genes |
| S | GW13_PRO3806 | | **3.00** | 1.67 | Uncharacterized conserved protein |
| S | GW13_PRO3802 | | **1.57** | 1.45 | YaeQ protein |
| S | GW13_PRO2895 | | 0.71 | **0.65** | Protein yifE |
| S | GW13_PRO2990 | | 4.60 | **0.64** | Protein of unknown function DUF414 |
| S | GW13_PRO3203 | | **1.89** | **0.42** | Protein yjbR |
| S | GW13_PRO0872 | | 1.72 | **0.42** | Cellobiose phosphotransferase system YdjC-like protein |
| S | GW13_PRO3443 | | 1.02 | **0.02** | hypothetical protein |
| T | GW13_PRO3261 | | **0.42** | 2.00 | Fumarate respiration sensor kinase protein DcuS |
| T | GW13_PRO4303 | | **2.33** | 1.20 | Osmosensitive K+ channel histidine kinase KdpD |
| T | GW13_PRO1364 | | **6.67** | 1.12 | Two-component sensor protein RcsD |
| U | GW13_PRO3689 | | **1.53** | 1.00 | Protein export cytoplasm protein SecA ATPase RNA helicase |
| U | GW13_PRO2572 | | **2.35** | 0.90 | Signal recognition particle receptor protein FtsY (alpha subunit) |
| V | GW13_PRO4055 | | **2.05** | 1.05 | RND efflux system, inner membrane transporter CmeB |
| V | GW13_PRO3215 | | 1.22 | **0.58** | Putative type-1 secretion protein |
| not found | GW13_PRO0175 | | n.a | **78.00** | gp8 |
| not found | GW13_PRO0176 | | n.a | **37.33** | hypothetical protein |
| not found | GW13_PRO1056 | | n.a | **20.67** | DNA-damage-inducible protein I |
| not found | GW13_PRO0152 | | n.a | **14.00** | c-type lectin precursor family member |
| not found | GW13_PRO0183 | | n.a | **12.67** | Phage protein |
| not found | GW13_PRO2297 | | 0.89 | **11.00** | hypothetical protein |
| not found | GW13_PRO0162 | | 0.83 | **8.00** | hypothetical protein |
| not found | GW13_PRO0165 | | n.a | **6.00** | Phage antitermination protein Q |
| not found | GW13_PRO0191 | | **2.33** | **6.00** | hypothetical protein |
| not found | GW13_PRO0227 | | 1.17 | **4.00** | hypothetical protein |
| not found | GW13_PRO4516 | | 0.67 | **4.00** | hypothetical protein |
| not found | GW13_PRO2774 | | **3.67** | 3.72 | probable secreted protein STY4010 |
| not found | GW13_PRO3290 | | 1.11 | **3.17** | Putative membrane protein |
| not found | GW13_PRO0212 | | **0.28** | 3.08 | Lysine-N-methylase |
| not found | GW13_PRO3278 | | n.a | **2.67** | hypothetical protein |
| not found | GW13_PRO0194 | | 0.83 | **1.83** | Phage tail length tape-measure protein 1 |
| not found | GW13_PRO4155 | | 1.14 | **1.83** | Phage tail fibers |
| not found | GW13_PRO1748 | | **0.39** | 1.50 | Putative transcriptional regulator |
| not found | GW13_PRO1926 | | **2.33** | 1.25 | CRISPR-associated protein, Cse1 family |
| not found | GW13_PRO2238 | | 0.81 | **0.50** | Inner membrane protein YqjK |
| not found | GW13_PRO1652 | | 1.33 | **0.30** | Transcriptional activator of cad operon |
| not found | GW13_PRO3444 | | **15.40** | **0.21** | DNA sulfur modification protein DndE |
| not found | GW13_PRO3447 | | **0.18** | **0.11** | DNA sulfur modification protein DndB |
| not found | GW13_PRO0312 | | **1.78** | n.a | hypothetical protein |
| ^a^Difference in expression of *dptB*ˉ and XTG102 compared to wild-type *S. enterica*; Values in red and blue color indicate proteins differentially expressed with a fold change of higher than 1.5 or lower than 0.66, respectively (p-value<0.05).  ^b^Data not available (due to low protein signal). | | | | | |

**
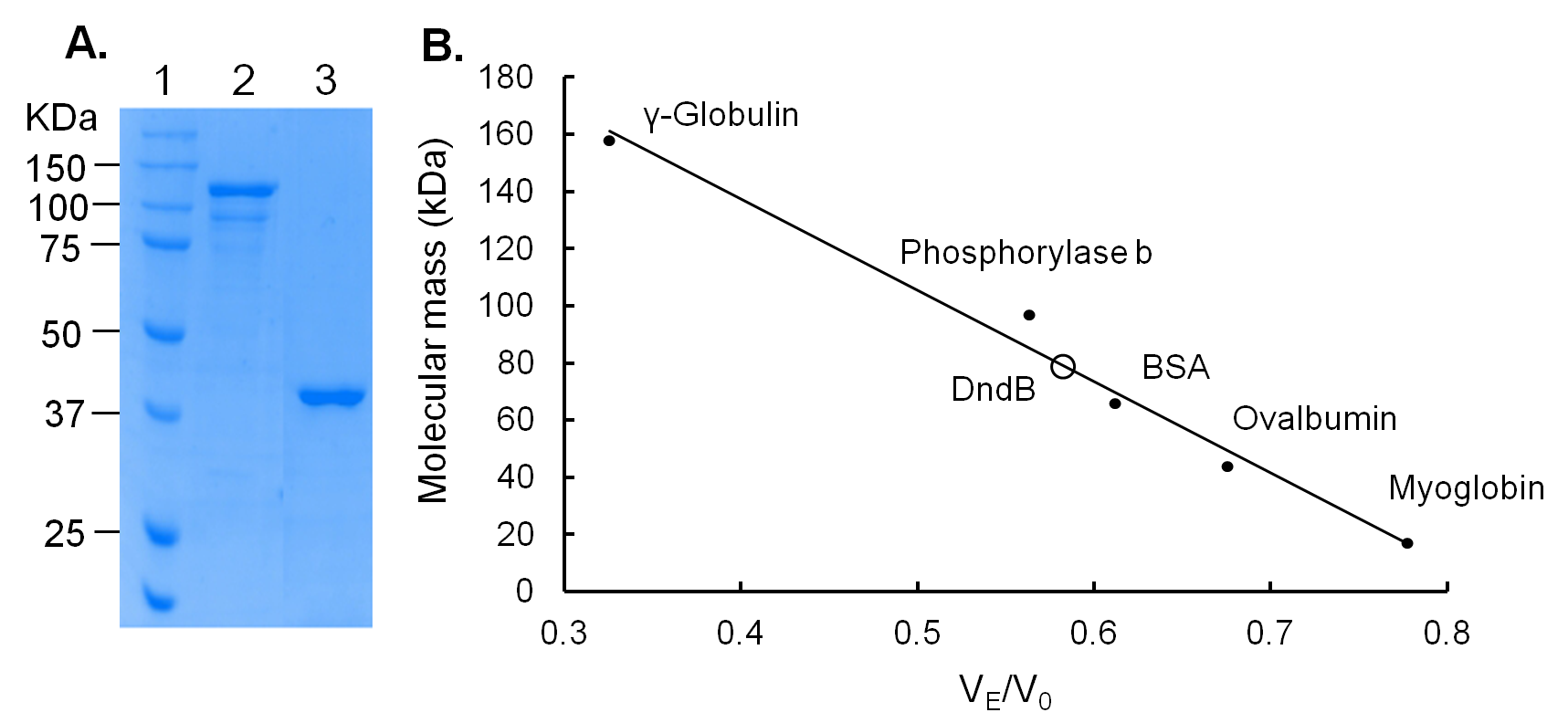
**

**Fig S1. Purification of *S. enterica* serovar Cerro 87 DndB and estimation of the native mass.** (A) SDS-PAGE gel showing purified DndB before and after treatment with TEV protease to cleave Nus-tag and His-tag (lanes 2 and lane 3, respectively); lane 1, molecular mass standards. (B) Size exclusion chromatography of DndB without tags using a HiLoad 16/60 Superdex 200 column. The native mass of DndB was estimated to be 79 kDa, suggesting that it exists as a homodimer. Calibration was conducted using myoglobin (17 kDa), ovalbumin (44 kDa), albumin (66 kDa), phosphorylase b (97 kDa), and γ-globulin (158 kDa). V_E_, elution volume of the protein; and V_0_, void volume of the column.

**
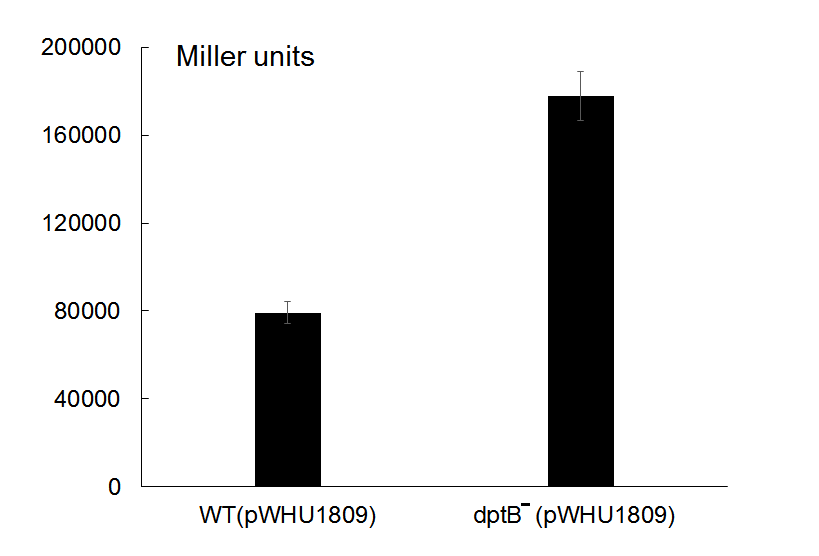
**

**Fig S2. β-Galactosidase activity assays.** Plasmid pWHU1809, carrying a transcriptional fusion of the B_1_ fragment with the *lacZ* reporter gene, was transformed into wild-type *S. enterica* serovar Cerro 87 and *dptB*ˉ strains. Gene expression was determined by measuring β-galactosidase activity. All experiments were performed in triplicate, and the mean values ± SD are indicated.
